# BRD4 Mediates Transforming Growth Factor-β-Induced Smooth Muscle Cell Differentiation from Mesenchymal Progenitor Cells

**DOI:** 10.3390/ijms26168074

**Published:** 2025-08-21

**Authors:** Ayobami Olajuyin, Venkatakirankumar Mandlem, Christudas Sunil, Yunzhuan Hou, Oluwaseun Adeyanju, Sana Petkar, Qinying Li, Torry A. Tucker, Steven Idell, Shi-You Chen, Xia Guo, Guoqing Qian

**Affiliations:** 1 Department of Cellular and Molecular Biology, The University of Texas Health Science Center at Tyler, Tyler, TX 75708, USA; amolajuy@utmb.edu (A.O.); venkatakirankumar.mandlem@uttyler.edu (V.M.); christudas.sunil@uthct.edu (C.S.); yunzhuan.hou@uthct.edu (Y.H.); oluwaseun.adeyanju@uthct.edu (O.A.); spetkar@uttyler.edu (S.P.); qinying.li@uthct.edu (Q.L.); torry.tucker@uthct.edu (T.A.T.); steven.idell@uthct.edu (S.I.); 2 Department of Surgery, School of Medicine, The University of Missouri, Columbia, MO 65211, USA; scqvd@missouri.edu

**Keywords:** smooth muscle cell, differentiation, TGF-β, BRD4, TAZ, myocardin

## Abstract

Smooth muscle cell (SMC) differentiation plays a crucial role in angiogenesis and vasculogenesis during embryonic development. The underlying mechanisms controlling SMC differentiation, especially progenitor-specific regulation, however, remain largely unclear. In this study, we identified bromodomain-containing protein 4 (BRD4) as a novel regulator for SMC differentiation. Transforming growth factor-β (TGF-β) induces BRD4 expression in the initial phase of SMC differentiation of pluripotent murine 10T1/2 cells. BRD4 was found critical in mediating TGF-β-induced SMC differentiation. Knockdown of BRD4 with siRNA suppressed TGF-β-induced expression of SMC markers including α-SMA and SM22α. In addition, the BRD4 inhibitor JQ1 and degraders ARV-825 and dBET1 suppressed TGF-β-induced SMC marker gene expression. BRD4 regulates SMC differentiation by activating SMC marker gene transcription. BRD4 mediated SMC differentiation is independent of the phosphorylation of Smad2/3. Instead, BRD4 mediated TAZ expression induced by TGF-β. Consistent with the function of TAZ, the inhibition of BRD4 reduced nuclear retention of Smad3, thereby impairing Smad3 mediated SMC gene transcription. Myocardin is an important transcriptional modulator for SMC markers. Interestingly, the knockdown of BRD4 also attenuated the induction of myocardin due to TGF-β in 10T1/2 cells. Taken together, this study demonstrates that BRD4 is a novel modulator for SMC differentiation from mesenchymal progenitor cells through the regulation of TAZ and myocardin.

## 1. Introduction

Smooth muscle cells (SMCs) are not terminally differentiated cells and adapt their phenotypes in response to various stimuli. The phenotypic switch of SMCs between differentiated and proliferative phenotypes is pathologically involved in several vascular diseases such as atherosclerosis, aortic aneurysm, hypertension, and restenosis [1,2]. Several types of stem cells can be induced to differentiate into SMCs, which include embryonic stem cells, mesenchymal stem cells, human-induced pluripotent stem cells, and adult stem cells. The differentiation towards SMCs is context-dependent [3,4,5,6], directed by growth factors (e.g., TGF-β and PDGF-BB), extracellular matrix stiffness, microRNAs, and others. Among them, TGF-β is a potent inducer of SMC differentiation. It signals through Smad-dependent and non-dependent pathways [6]. In addition, Smads can also be regulated by transcription factors or modulators to fine-tune the process, including, e.g., meox-1 [7], RhoA [8], myocardin [9], etc. Despite the continuing efforts, the detailed mechanisms remain not fully understood.

Bromodomain containing protein 4 (BRD4) is a member of the bromodomain and extra-terminal domain (BET) family of proteins. In addition to BRD4, there are three other members of the BET family, BRD2, BRD3, and BRDT. BRD4 is the most studied member. BRD4 is a crucial epigenetic regulator that modulates diverse cellular processes important for inflammation, differentiation, and apoptosis [10,11]. Their function is mediated by the binding to acetylated histone and other nuclear proteins, including transcription factors, (super)enhancers, adaptor proteins, etc. BET inhibitors mitigate the differentiation into cardiomyocytes from mouse embryonic stem cells (mESC) or human induced pluripotent stem cells (hiPSC)-derived cardiac progenitor cells (CPCs). Deletion of the BRD4 gene results in embryonic or early postnatal lethality in mice with myocardial hypoplasia and an increase in CPCs [12]. BRD4 has also been reported to mediate osteogenic cell differentiation [13] and hematopoietic differentiation [14] from human bone marrow mesenchymal stem cells and human embryonic stem cells, respectively. It also regulates neural crest differentiation into smooth muscle [15]. The interaction of BRD4 and TGF-β signaling has been reported in different cell types, including epithelial cells, fibroblasts, cardiomyocytes, etc. In addition, BRD4 also mediates TGF-β-induced fibroblast activation in the context of cardiac fibrosis [16,17] and pulmonary fibrosis [18]. It remains unclear whether BRD4 plays a role in SMC differentiation from mesenchymal stem cells. To further understand the regulating mechanism of SMC differentiation, we investigated whether BRD4 is a novel mediator of this process and a possible target for modulating SMC differentiation using the C3H/10T1/2 (10T1/2) cell model.

In our study, we found that BRD4 mediates TGF-β-induced differentiation of mouse mesenchymal stem cells into SMCs. Knockdown of BRD4 attenuated the expression of SMC markers smooth muscle cell alpha-actin (α-SMA) and smooth muscle protein 22-alpha (SM22α). Mechanistically, we observed that BRD4 mediates the expression of TAZ and myocardin to facilitate the expression of these SMC markers. The findings suggest that BRD4 is a potential target for SMC differentiation and may have implications in SMC phenotype-related vascular diseases.

## 2. Results

### 2.1. BRD4 Expression Is Up-Regulated in TGF-β-Induced SMC Differentiation

**Figure 1:**
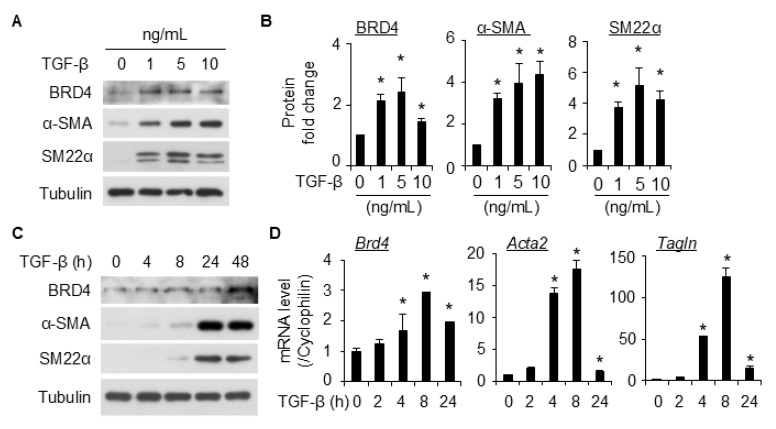
TGF-β-induced BRD4 expression along with SMC markers in 10T1/2 cells. (**A**,**B**), TGF-β-induced BRD4 and SMC marker expression dose dependently in 10T1/2 cells. Serum-starved 10T1/2 cells were treated with vehicle or different concentrations of TGF-β as indicated for 48 h. BRD4 and VSMC markers (α-SMA and SM22α) were detected by Western blotting (WB, (**A**)) and quantified in (**B**). Tubulin served as the loading control. *, *p* < 0.05 compared to vehicle group (0 ng/mL, (**B**)), n = 3 independent experiments. (**C**,**D**), 10T1/2 cells were starved for 24 h, followed by vehicle or TGF-β (5 ng/mL) induction for various times as indicated. WB (**C**) and qPCR (**D**) were performed to detect BRD4 and SMC marker protein and mRNA expression, respectively. *, *p* < 0.05 compared to vehicle group (0 h, (**D**)), n = 3 replicates. Cyclophilin was the internal control for qPCR.

10T1/2 cells are a well-established model to study SMC differentiation from stem cells [19]. Upon TGF-β treatment, 10T1/2 cells express markers of SMCs, including α-SMA and SM22α at both the protein (Figure 1A–C) and mRNA (Figure 1D) levels. TGF-β potently induced these markers’ expression along with BRD4 at all the doses tested (1–10 ng/mL, Figure 1A). The dose of TGF-β at 5 ng/mL sufficiently induced the cell differentiation and BRD4 expression, and a higher dose did not induce further increase; therefore, it was used for subsequent experiments. Notably, BRD4 expression was significantly induced as early as 4 h following TGF-β treatment (Figure 1C), suggesting that BRD4 might be involved in TGF-β-induced SMC differentiation.

### 2.2. BRD4 Is Involved in TGF-β-Induced SMC Differentiation

**Figure 2:**
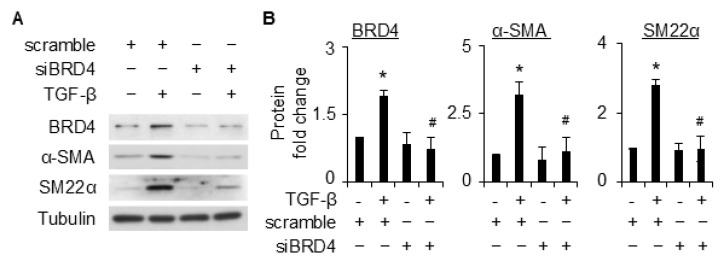
BRD4 knockdown attenuated TGF-β-induced differentiation of 10T1/2 cells to SMCs. (**A**), 10T1/2 cells were transfected with scramble or siBRD4, followed by treatment with vehicle or TGF-β (5 ng/mL) for an additional 48 h. The expression of BRD4 and SMC markers was detected by WB (**A**). (**B**) is the quantification of A based on 3 independent experiments. *, *p* < 0.05 compared to vehicle-treated scramble; #, *p* < 0.05 compared to TGF-β-treated scramble.

To determine if BRD4 is involved in TGF-β-induced SMC differentiation, we knocked down BRD4 expression with siRNA, followed by TGF-β induction to detect SMC marker expression. BRD4 and SMC markers were induced simultaneously by TGF-β, and BRD4 induction was remarkably blocked by its targeting siRNA (Figure 2A,B). Interestingly, knockdown of BRD4 significantly diminished TGF-β-induced expression of SMC markers α-SMA and SM22α (Figure 2A,B). BRD4 knockdown appeared not to alter the basal level expression of SMC markers. These results showed that BRD4 is essential for TGF-β-induced SMC differentiation in 10T1/2 cells.

**Figure 3:**
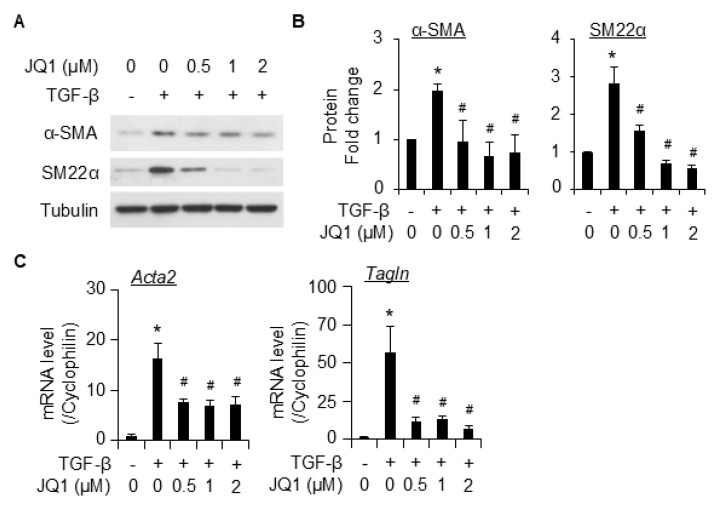
BRD4 inhibitor JQ1 inhibited TGF-β-induced differentiation of 10T1/2 cells into SMCs. (**A**,**B**), serum-starved 10T1/2 cells were pretreated with different doses of JQ1, followed by TGF-β treatment for an additional 48 h, and cells were then harvested for WB analysis of SMC markers. Tubulin was used as the loading control. B is the quantification of A based on 3 independent experiments. *, *p* < 0.05 compared to vehicle control; #, *p* < 0.05 compared to TGF-β treatment alone group. (**C**), Serum-starved 10T1/2 cells were pretreated with different doses of JQ1, followed by TGF-β treatment for an additional 16 h before cells were harvested for qPCR analysis of SMC markers. Cyclophilin was used as an internal control. *, *p* < 0.05 compared to vehicle control; #, *p* < 0.05 compared to TGF-β treatment alone group. n = 3 replicates.

**Figure 4:**
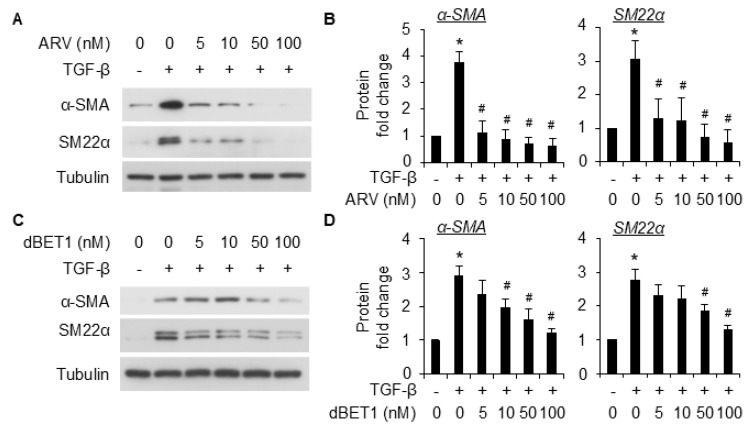
BRD4 degraders inhibited TGF-β-induced differentiation of 10T1/2 cells into SMCs. (**A**,**C**), Serum-starved 10T1/2 cells were pretreated with different doses of ARV-825 (ARV, (**A**)) or dBET1 (**C**), followed by TGF-β treatment (5 ng/mL) for an additional 48 h, and the cells were then harvested for WB analysis of SMC markers. Tubulin was used as the loading control. (**B**,**D**) are the quantification of (**A**,**C**), respectively, based on 3 independent experiments. *, *p* < 0.05 compared to vehicle control; #, *p* < 0.05 compared to TGF-β treatment alone group.

We next evaluated the effect of pharmaceutical BRD4 inhibitor (JQ1) and degraders (ARV-825 and dBET1) on TGF-β-induced SMC differentiation. Pretreatment with JQ1 significantly inhibited the induction of SMC markers α-SMA and SM22α at both protein (Figure 3A,B) and mRNA (Figure 3C) levels. The degraders AVR-825 (Figure 4A,B) and dBET1 (Figure 4C,D) inhibited SMC marker expression caused by TGF-β in a dose-dependent manner. Among the degraders, ARV-825 appears to be more effective than dBET1; however, both degraders were more effective than JQ1 in suppressing SMC differentiation from 10T1/2 cells. No effect on cell viability was observed microscopically even at the highest dose tested for each inhibitor/degrader using trypan blue staining.

### 2.3. BRD4 Mediated SMC Maker Expression Appears to Be Independent of Smad3 Phosphorylation

Smad3 phosphorylation and nuclear translocation are critical for transcriptional regulation of SMC marker genes [20,21]. To explore the mechanism underlying BRD4-mediated SMC differentiation maker expression, we tested whether BRD4 affects Smad2/3 activation. The results show that JQ1 treatment did not dramatically affect Smad2/3 phosphorylation (Supplemental Figure S1), suggesting that the effect of BRD4 in mediating SMC maker expression is Smad3 phosphorylation independent.

### 2.4. BRD4 Mediates SMC Marker Gene Expression Through TAZ

**Figure 5:**
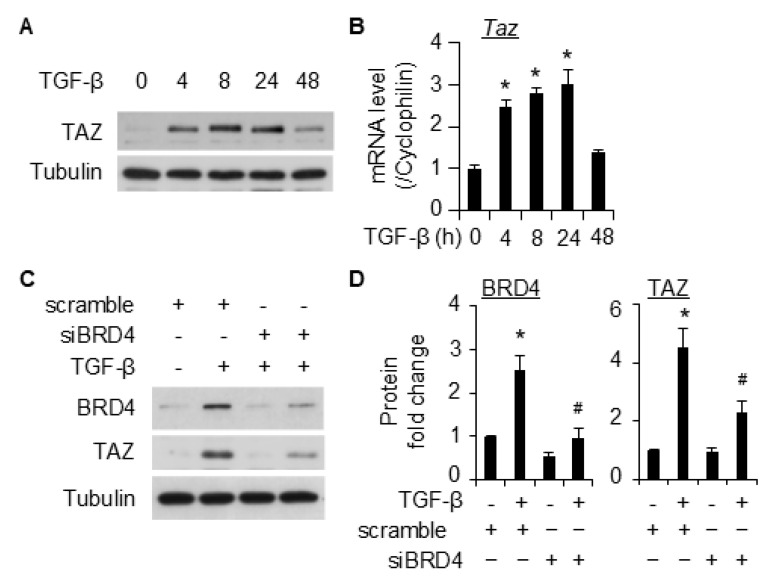
BRD4 knockdown inhibited TGF-β-induced TAZ expression in 10T1/2 cells. (**A**,**B**), Serum-starved 10T1/2 cells were treated with TGF-β treatment (5 ng/mL) for different times as indicated before the cells were harvested for WB (**A**) and qPCR (**B**) analysis of TAZ. Tubulin was used as the loading control. Cyclophilin was used as the internal control for qPCR analysis in B. *, *p* < 0.05 compared to control (0 h), n = 3 replicates. (**C**), 10T1/2 cells were transfected with scramble control or siBRD4, followed by treatment with vehicle or TGF-β (5 ng/mL) for an additional 8 h. The cells were then harvested for analysis of given protein by WB. (**D**) is the quantification of C based on 3 independent experiments. *, *p* < 0.05 compared to the vehicle-treated scramble; #, *p* < 0.05 compared to TGF-β-treated scramble.

**Figure 6:**
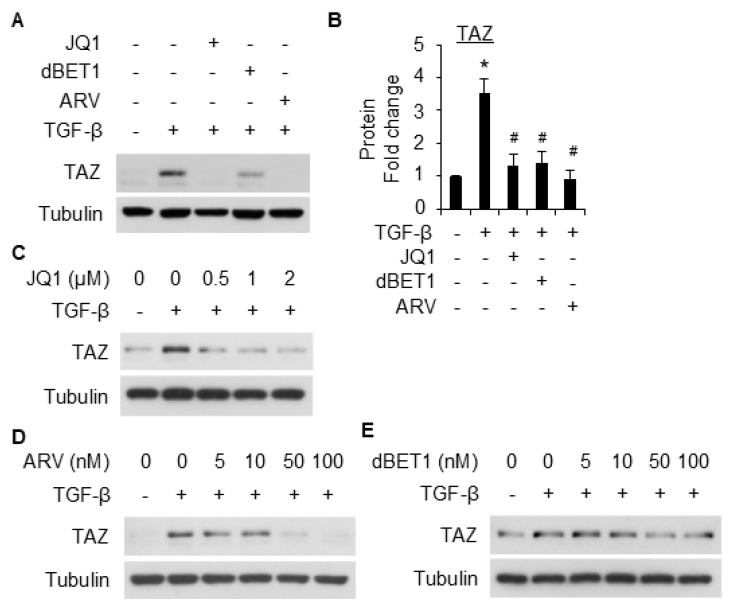
BRD4 inhibitors suppressed TGF-β-induced TAZ expression in 10T1/2 cells. (**A**), serum-starved 10T1/2 cells were pretreated with JQ1 (2 µM), dBET1 (100 nM), or ARV-825 (ARV, 100 nM), followed by TGF-β treatment (5 ng/mL) for an additional 8 h. The cells were then harvested for WB analysis of TAZ. Tubulin was used as the loading control. (**B**) is the quantification of A based on 3 independent experiments. *, *p* < 0.05 compared with vehicle control; #, *p* < 0.05 compared to TGF-β treatment alone group. (**C**–**E**), serum-starved 10T1/2 cells were pretreated with different doses of JQ1 (**C**), ARV-825 (ARV, (**D**)) or dBET1 (**E**), followed by TGF-β treatment (5 ng/mL) for an additional 8 h to detect TAZ through WB.

TAZ regulates Smad3 nucleocytoplasmic shuttling, resulting in nuclear accumulation of Smad3 [22]. Silencing TAZ affects SMC marker gene expression [23]. We therefore hypothesized that TAZ is involved in BRD4-mediated SMC gene expression. Consistent with previous reports [23,24], TGF-β induced TAZ expression at both the protein (Figure 5A) and the RNA (Figure 5B) levels. Importantly, the knockdown of BRD4 with siRNA significantly attenuated the induction of TAZ by TGF-β in 10T1/2 cells (Figure 5C,D). To further test this hypothesis, different inhibitors were used to block BRD4. Pretreatment of 10T1/2 cells with either JQ1, ARV-825, or dBET1 significantly suppressed the induction of TAZ by TGF-β (Figure 6A,B). Further dose–response studies confirmed that all three inhibitors suppressed TGF-β-induced TAZ expression in a dose-dependent manner in 10T1/2 cells (Figure 6C–E). These results suggest that BRD4 may affect SMC marker gene expression by regulating TAZ.

### 2.5. Myocardin Is Involved in BRD4-Mediated SMC Marker Gene Expression

**Figure 7:**
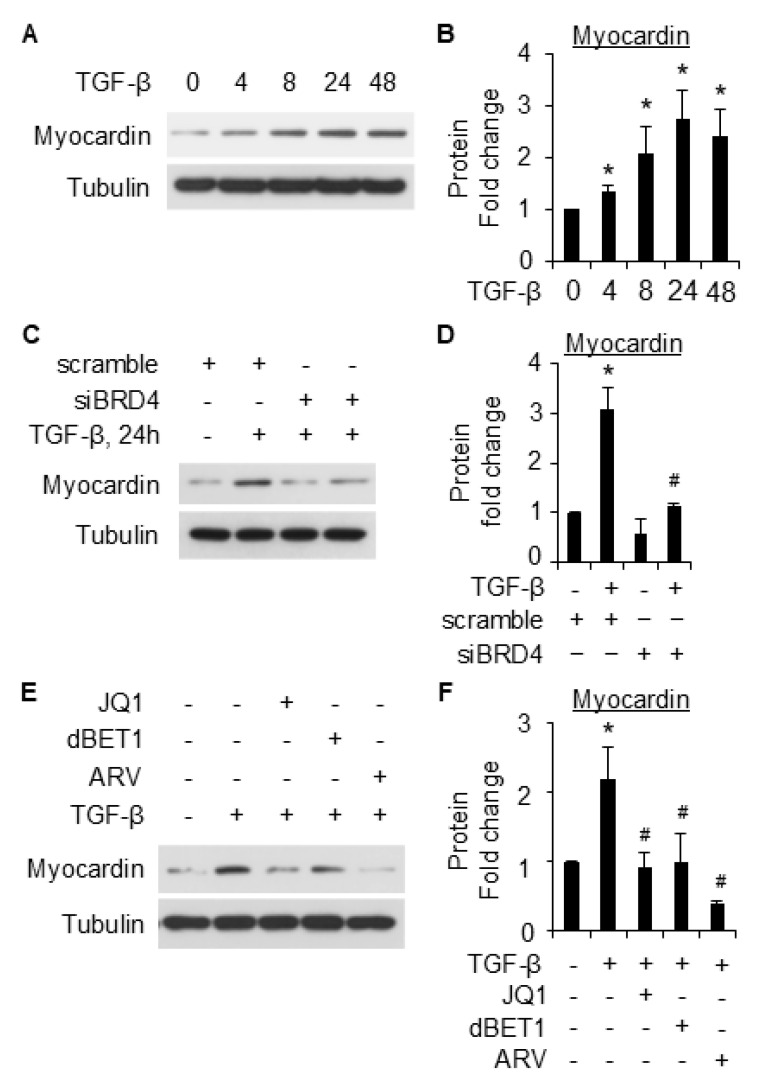
Myocardin is involved in BRD4 mediated10T1/2 differentiation into SMCs. (**A**) Serum-starved 10T1/2 cells were treated with TGF-β (5 ng/mL) for various times to detect Myocardin expression through WB. (**B**) is the quantification of A based on three independent experiments. *, *p* < 0.05 compared with control (0 h). (**C**) 10T1/2 cells were transfected with scramble control or siBRD4, followed by treatment with vehicle or TGF-β (5 ng/mL) for an additional 24 h. Myocardin expression was detected through WB. (**D**) is the quantification of C based on three independent experiments. *, *p* < 0.05 compared with the vehicle-treated scramble; #, *p* < 0.05 compared with TGF-β-treated scramble. (**E**) serum-starved 10T1/2 cells were pretreated with JQ1 (2 µM), dBET1 (100 nM), or ARV-825 (100 nM), followed by TGF-β treatment (5 ng/mL) for an additional 24 h. The cells were then harvested for WB analysis of myocardin. (**F**) is the quantification of E based on three independent experiments. *, *p* < 0.05 compared to vehicle control; #, *p* < 0.05 compared to TGF-β treatment alone group.

Myocardin is a transcriptional coactivator that is known to regulate SMC gene expression [25]. To further explore the underlying mechanisms underlying BRD4-mediated SMC gene expression, we tested whether BRD4 regulates myocardin to upregulate SMC marker gene expression in 10T1/2 cells. TGF-β induced myocardin expression in 10T1/2 cells in a time-dependent manner, as early as 4 h after treatment (Figure 7A,B). Intriguingly, the knockdown of BRD4 significantly reduced the induction of myocardin by TGF-β (Figure 7C,D). BRD4 inhibitors (JQ1, ARV-825, and dBET1) all significantly suppressed TGF-β-induced upregulation of myocardin, although at varying levels (Figure 7E,F). The data suggest that the effect of BRD4 in mediating SMC gene expression might be partly accounted for by myocardin.

### 2.6. BRD4 Enhances Smad3 Nuclear Retention Likely Involving TAZ

**Figure 8:**
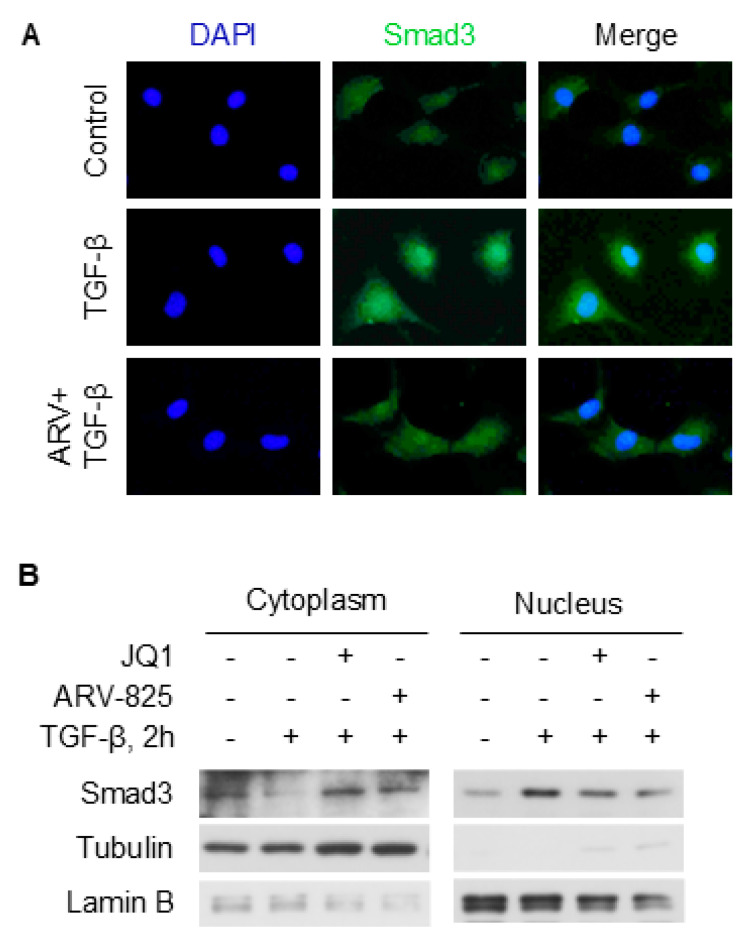
BRD4 inhibition reduced the nuclear levels of Smad3 induced by TGF-β in 10T1/2 cells. (**A**) Serum-starved 10T1/2 cells were pretreated with ARV-825 (100 nM) for 30 min, followed by TGF-β treatment (5 ng/mL) for an additional 2 h, and cells were then subject to immunofluorescence staining of Smad3. Images were taken at 200×. (**B**) Serum-starved 10T1/2 cells were pretreated with JQ1 (1 µM) or ARV-825 (100 nM), followed by TGF-β treatment (5 ng/mL) for an additional 2 h, and cells were then subject to fractionation WB analysis of Smad3. Tubulin and Lamin B1 were used as loading controls for the cytoplasm and nucleus, respectively.

Smad3 nuclear translocation is critical for its transcriptional regulation of SMC marker genes. TAZ regulates Smad3 nuclear retention in 10T1/2 cells [24]. Since BRD4 enhances TAZ expression, we tested whether inhibition of BRD4 affects Smad3 subcellular localization through fractionation WB. Immunofluorescence staining showed that nuclear Smad3 was notably increased by TGF-β after 2 h treatment (Figure 8A). In contrast, ARV-825 attenuated the nuclear accumulation of Smad3. Further, TGF-β treatment reduced cytoplasmic Smad3 while increasing its nuclear level in 10T1/2 cells. Interestingly, both JQ1 and ARV-825 pretreatment attenuated such effects induced by TGF-β (Figure 8B). Taken together, these data suggest that BRD4 might affect Smad3 nuclear retention, likely through TAZ.

## 3. Discussion

The differentiation from progenitor cells towards SMCs is under complex and precise regulation. Using an established in vitro model, we identified BRD4 as a novel factor to mediate SMC differentiation from mouse progenitor cells. We found that BRD4 blockage by siRNA or inhibitors mitigated SMC differentiation. Further, BRD4 was found to increase TAZ levels, to increase Smad3 nuclear retention and enhance myocardin expression to facilitate SMC marker gene expression. These findings provide novel insights about the regulation of SMC differentiation and may have implications in relevant human diseases.

TGF-β is a potent stimulator for SMC differentiation from the mouse pronator cell 10T1/2. Various lines of evidence show the interaction of TGF-β signaling with BRD4 [17,26], implying BRD4 as a critical modulator for the TGF-β signaling. In this study, we provided proof of concept that BRD4 is an important modulator for SMC differentiation. We found that BRD4 was induced by TGF-β in 10T1/2 cells (Figure 1). An early induction of BRD4 by TGF-β (2 h post-treatment) suggests that it might be involved in the early changes during the differentiation of 10T1/2 towards SMCs. Indeed, knockdown of Brd4 significantly attenuated TGF-β-induced SMC marker gene expression (Figure 2). Pretreatment of 10T1/2 cells with JQ1 dose-dependently inhibited SMC marker expression induced by TGF-β at both protein (Figure 3A,B) and mRNA (Figure 3C) levels. Further, the BET degraders ARV-825 and dBET1 also inhibited SMC maker expressions induced by TGF-β at much lower doses than JQ1 (Figure 4). These two degraders are more effective than the inhibitor JQ1. The data suggests the involvement of BRD4 in the transcriptional regulation of SMC markers.

BRD4 is an important regulator of gene expression that is involved in many cellular functions. It has been increasingly reported to interact with TGF-β/Smad3 signaling. For instance, TGF-β induces BRD4 expression in human [27] and rat [17] fibroblasts. BRD4 was also reported to bind to Smad3 to enhance its phosphorylation in human epithelial cell line HK-2 cells [28]. In another study using the prostate cancer cell line DU145, pretreatment with the BRD3 degrader MZ1 did not affect Smad3 phosphorylation induced by TGF-β [29]. It appears that a cell-dependent effect exists regarding the interaction between BRD4 and Smad3. In this study, we failed to observe the effect of BRD4 inhibition on phosphorylation of either Smad2 or Smad3 (Supplemental Figure S1). In spite of this, we found that the inhibition of BRD4 decreased nuclear Smad3 levels induced by TGF-β (Figure 8). This discrepancy could be due to the modulation of some factors that could retain Smad3 in the nucleus. TAZ has been reported to increase the nuclear translocation of Smad3 and positively regulate SMC marker gene expression [23]. Therefore, we tested whether BRD4 regulates TAZ to increase the nuclear Smad3 level. Consistent with previous reports in other cell types [30,31], TGF-β induced the expression of TAZ in 10T1/2 cells (Figure 5). Interestingly, TAZ was found to be regulated by BRD4 since siRNA and inhibitors of BRD4 both attenuated TGF-β-induced TAZ expression. The data demonstrates that BRD4 may regulate SMC differentiation through the modulation of TAZ. Whether the mechanism holds in SMC differentiation from other stem cells or under different stimuli instead of TGF-β is worth further study.

Myocardin is a key transcriptional coactivator that regulates gene expression through binding to and activation of serum response factor (SRF) [32]. The latter regulates cell differentiation through binding to the CArG cis elements within almost all the SMC-specific promoters and can be induced by TGF-β [33,34]. In this study, we found that TGF-β induced myocardin expression during SMC differentiation from 10T1/2 cells (Figure 7A). Importantly, the knockdown of BRD4 notably suppressed myocardin expression induced by TGF-β (Figure 7C,D). The inhibition of BRD4 with different inhibitors reduced the induction of myocardin by TGF-β (Figure 7E,F). These data suggest that BRD4 may regulate SMC differentiation through myocardin. A previous study showed that BRD4 inhibition downregulates myocardin-related transcription factor A (MRTF-A) [35]. Our findings add novel knowledge about the BRD4-myocardin axis in cell differentiation regulation. Further exploring the detailed regulating mechanism and its role in the pathogenesis of SMC-related diseases might be of interest. In addition, there are a few limitations of the study. First, the current study used murine cell lines, and the use of human stem cells or ex vivo tissues will strengthen the significance of the study. Second, since BRD4 has both long and short isoforms, whether these two isoforms play similar or different roles towards SMC differentiation remains an interesting yet unanswered question. The antibody used in this study only recognizes the long isoform, and whether the short isoform plays a role in this context could be an extension of this current work. Previous studies showed that the long and short isoforms of BRD4 act differently in tumor progression and metastasis [36,37]. It is likely that the BRD4 short isoform may have its distinct effects on SMC differentiation from 10T1/2 cells, which warrants further investigation.

Taken together, this study uncovered a previously unrecognized role of BRD4 in SMC differentiation from mouse progenitor cells. BRD4 regulates myocardin and TAZ expression to mediate the expression of smooth muscle cell marker genes. These novel observations warrant further testing in different contexts of SMC differentiation, especially in VSMC-related cardiovascular diseases. The findings from this study provide novel insight into the regulation of SMC differentiation by BRD4 and suggest that BRD4 might serve as a target for SMC differentiation-related diseases.

## 4. Materials and Methods

### 4.1. Reagents and Antibodies

Recombinant human transforming growth factor-β1 (TGF-β, 7754-BH-025/CF) was purchased from R&D Systems, Inc. (Minneapolis, MN, USA). The small molecular inhibitor of BRD4 JQ1 (HY-13030) and degraders ARV-825 (HY-16954) and dBET1 (HY-101838) were purchased from MedChemExpress (Monmouth Junction, NJ, USA). The primary antibodies used in the study includes BRD4 (A310-985A-T) from Bethyl Laboratories Inc. (Montgomery, TX, USA); TAZ (sc-518026) and Lamin B1 (SC-374015) from Santa Cruz Technology (Dallas, TX, USA); myocardin (ab22073) and SM22α (ab10135) from Abcam (Cambridge, UK); Smad3 (9523S), Smad2 (5339S), phosphor-Smad2 (3108S), and phosphor-Smad2 (3108S) from Cell Signaling Technology (Danvers, MA, USA); α-SMA (clone 1A4) and Tubulin (T5168) from Sigma-Aldrich (St. Louis, MO, USA).

### 4.2. Cell Culture and Treatments

C3H/10T1/2 (10T1/2, clone CCL-226) cells were purchased from ATCC (Manassas, VA, USA) and cultured in Dulbecco’s Modified Eagle’s Medium (DMEM) containing 10% fetal bovine serum (FBS) and 5% L-Glutamine and supplemented with penicillin/streptomycin (1:100). The cells were starved in serum-free DMEM for 24 to 48 h prior to inhibitor and/or TGF-β treatment.

### 4.3. Western Blotting (WB) and Fractionation WB Assays

The WB assay was performed as previously described [38,39]. Briefly, whole cell lysates were collected from the cells after treatments in radioimmunoprecipitation assay (RIPA) lysis buffer containing proteinase inhibitor cocktail and phosphatase inhibitor (ThermoFisher Scientific, Waltham, MA, USA). The bicinchoninic acid (BCA) protein assay kit (ThermoFisher Scientific) was used to measure the total protein concentration according to the manufacturer’s guide. Following that, the protein samples were denatured with SDS by heating and subjected to SDS-PAGE gel electrophoresis and transferred to PVDF membranes. The membranes were blocked with 5% (*w*/*v*) non-fat milk in PBST and incubated with primary antibodies at 4 °C overnight with gentle shaking. Secondary HRP-conjugated antibodies were used to detect given proteins, including anti-rabbit IgG-HRP (31460) and anti-mouse IgG-HRP (31430) that were purchased from ThermoFisher Scientific. The subcellular analysis of Smad3 was performed using the NE-PER™ Nuclear and Cytoplasmic Extraction Reagents (78833) from ThermoFisher Scientific following the manufacturer’s guide.

### 4.4. RNA Extraction and Quantitative Real-Time PCR (qPCR) Analysis

Total RNA was extracted from the cells after treatment using the TRIzol reagent according to the manufacturer’s guide. After quantification of RNA concentrations, total RNA samples (1 µg) were used for reverse transcription to produce cDNA templates using a reverse transcription kit from BioRad (Hercules, CA, USA). Diluted cDNA templates were then used for qPCR analysis of target genes using the SYBR reagent (BioRad). Cyclophilin A was used as an internal control. The primers for probing the given genes were reported previously [7] and shown in Supplementary Table S1.

### 4.5. Small Interfering RNA (siRNA) Transfection

To knock down the expression of BRD4 in 10T1/2 cells, the control scramble (SIC001, Sigma, Burlington, MA, USA) and siRNA targeting BRD4 (siBRD4, SASI_Mm01_0011632, Sigma) were used. Cells were seeded in 6 cm dishes at a confluence of approximately 50% before transfection with scrambling or siBRD4 using the jetPRIME from Polyplus transfection (New York, NY, USA) according to the manufacturer’s guide. Cells were then starved in serum-free DMEM overnight prior to the treatment with vehicle or TGF-β.

### 4.6. Immunofluorescence Staining

Immunofluorescence staining was performed similarly as previously reported [40,41]. After treatment, the cells seeded on cover slides were washed with 1xPBS, then fixed with 10% formaldehyde for 10 min at room temperature (RT). After washing three times with PBST, the cells were permeabilized with 0.1% Triton X-100 in PBST for 10 min at RT, followed by three times washing with PBST. The cells were then blocked with 10% goat serum in PBS for 15 min at RT, followed by incubation with anti-Smad3 antibody overnight at 4 °C. Next, the cells were briefly washed with PBST, followed by incubation with a secondary Alexa Fluor Plus 488 conjugated antibody (A32731, ThermoFisher Scientific) for 1 h at RT. The cells were then mounted with anti-fade DAPI and kept in the dark until imaging using a Nikon NiU microscope (Nikon Instruments Inc., Melville, NY, USA).

### 4.7. Statistical Analysis 

The data shown in this study were presented as means ± SD. The comparisons among different groups were performed using one-way analysis of variance (ANOVA) followed by Dunnett’s test. The software GraphPad Prism 10 was used for statistical tests. A *p*-value of less than 0.05 was considered statistically significant.

## Data Availability

The data presented in this study are available upon request.

## Supplementary Materials

The following supporting information can be downloaded at: https://www.mdpi.com/article/10.3390/ijms26168074/s1.

## Abbreviations

The following abbreviations are used in this manuscript:

BRD4	bromodomain-containing protein 4
BCA	bicinchoninic acid
IF	immunofluorescence
RIPA	radioimmunoprecipitation assay
TGF-β	transforming growth factor-β
SMC	smooth muscle cell
TAZ	transcriptional coactivator with PDZ-binding motif
