# Supplementary material for: BRD4 Mediates Transforming Growth Factor-β-Induced Smooth Muscle Cell Differentiation from Mesenchymal Progenitor Cells
Source: Int J Mol Sci. 2025 Aug 21;26(16):8074. doi: 10.3390/ijms26168074 (PMC12386208; doi:10.3390/ijms26168074)
Supplement: Supplementary file 1 [file ijms-26-08074-s001.zip › ijms-3808498-supplementary.pdf]

Supplementary materials:

Supplemental Table S1. qPCR primers used in the study.

Genes		Primer sequences
Brd4	Forward	5’-GCCATCTACACTACGAGAGTTGG-3’
	Reverse	5’-ATTCGCTGGTGCTCTCCGACTC-3’
Acta2	Forward	5'-AATGGCTCTGGGCTCTGTAAG-3'
	Reverse	5'-CACGATGGATGGGAAAACAGC-3'
Tagln	Forward	5'-GGTCCATCCTACGGCATGAG-3'
	Reverse	5'-CCTACATCAGGGCCACACTG-3'
Taz	Forward	5'-TGCTACAGTGTCCCCACAAC-3'
	Reverse	5'-TGACCGGAATTTTCACCTGT-3'
Ppia	Forward	5'-GAGCTGTTTGCAGACAAAGTTC-3'
	Reverse	5'-CCCTGGCACATGAATCCTGG-3'

Supplementary Figure S1

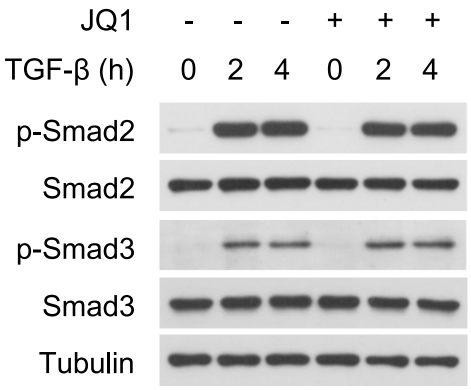

Supplementary Figure S1. Inhibition of BRD4 with JQ1 did not affect the phosphorylation of Smads in response to TGF-β in 10T1/2 cells. Serum-starved 10T1/2 cells were pretreated with or without JQ1 (1 μM) for 30 min, followed by TGF-β (5 ng/mL) treatment for an additional 2 and 4 h, respectively. The cells were then harvested for Western blotting analysis of phosphor(p)-Smad2, p-Smad3, and total Smad2 and Smad3. Tubulin is the loading control.
